# Examination of Lower Level Motion Mechanisms That Provide Information to Object Tracking: An Examination Using Dichoptic Stimulation

**DOI:** 10.1177/2041669519891745

**Published:** 2019-11-28

**Authors:** Hidetoshi K. Kanaya, Marie M. Morita, Takao Sato

**Affiliations:** College of Comprehensive Psychology, Ritsumeikan University, Osaka, Japan; Department of Psychology, Ritsumeikan University, Kyoto, Japan; Japan Society for the Promotion of Science, Tokyo, Japan; College of Comprehensive Psychology, Ritsumeikan University, Osaka, Japan

**Keywords:** object tracking, apparent motion, dichoptic, first-order motion, second-order motion

## Abstract

In this study, we examined the operation of first- and second-order motion mechanisms with respect to object tracking using dichoptic presentation. A bistable apparent motion stimulus composed of four rectangles arranged in square- and diamond-shapes in every other frame was presented binocularly, monocularly, or dichoptically using a stereoscope. Since past motion studies showed that the first-order motion mechanism cannot function under dichoptic stimulation, we evaluated the upper temporal frequency limits of object tracking with dichoptic presentation and compared these results with those obtained with ordinary binocular or monocular (nondichoptic) presentation. We found that the temporal limits were 4 -5 Hz, regardless of the viewing conditions. These limits are similar to those for within-attribute (first- and second-order) object tracking (4 -5 Hz) obtained in our previous study. Thus, this putative mechanism may be responsible for object tracking, based only on second-order components, even in the case of first-order stimuli.

## Introduction

Kanaya and Sato (2012) investigated the temporal characteristics of object tracking (e.g., Verstraten, Cavanagh, & Labianca, 2000) using bistable apparent motion stimuli defined by the same or different visual attributes (within- or cross-attribute motion), such as luminance, motion, binocular disparity, flicker, and contrast. The main objective of the study was to identify visual processes that mediate object tracking by comparing upper temporal limits of object tracking that use within- or cross-attribute motions. Bistable apparent motion stimuli generated by frames each defined by different attributes are not detected by lower level motion mechanisms but detected by a complicated, higher order processes in which each attribute stimulus is allocated on a saliency map by selecting a salient feature using attention (e.g., Lu & Sperling, 1995a, 2001). On the other hand, within-attribute motion can be detected by lower level motion mechanisms such as first- or second-order motion mechanisms. Their results can be summarized in the following two points. First, the upper temporal limits of object tracking for within-attribute motion ranged between 4 and 5 Hz. This is much higher than the 2 to 3 Hz limits of object tracking for cross-attribute motion that corresponds to the limit of voluntary shift of attention reported in Kanaya and Sato (2012). Second, with respect to object tracking using within-attribute motion, upper temporal limits for first-order stimuli were lower than that for first-order motion detection and were much the same as those for second-order stimuli.

Their first results suggest that within-attribute object tracking depends more on lower level motion information than on attention, at least for the stimulus with temporal frequency above 3 Hz. These results agree with several past object tracking studies (e.g., Huff & Papenmeier, 2013; St.Clair, Huff, & Seiffert, 2010), reporting that motion information contributes to object tracking, although they used a different paradigm (multiple object tracking).

On the other hand, their second results seem to be counterintuitive. Past motion studies have shown that the temporal characteristics of motion detection for first-order (luminance-defined) motion are quite different from those for several types of second-order motions based on second-order statistics, such as contrast, texture, motion, binocular disparity, and flicker. Observers are sensitive to first-order motions at high temporal frequencies, that is, they can detect motion at temporal frequencies up to several tens of hertz (e.g., Burr & Ross, 1982; Lu & Sperling, 1995b), while they are insensitive to second-order motions at high temporal frequencies beyond several Hz (e.g., Hutchinson & Ledgeway, 2006; Lu & Sperling, 1995b, 2001; Smith & Ledgeway, 1998).^1^ This difference can be attributed to the difference in temporal characteristics of mechanisms detecting first- and second-order motions. For luminance-defined dot patterns or grating stimuli, motion is thought to be detected by the putative first-order motion detectors composed of combinations of spatiotemporal filters (e.g., Adelson & Bergen, 1985; van Santen & Sperling, 1985; Watson & Ahumada, 1985). On the other hand, it has been suggested that there are different mechanisms for detecting second-order motion (e.g., Cavanagh & Mather, 1989; Lu & Sperling, 1995b, 2001). However, contrast-defined motion can be detected by a much lower level mechanism similar to that for first-order motion with a simple preprocessing (e.g., Lu & Sperling, 1995b, 2001). However, the results reported by Kanaya and Sato (2012) were different from these tendencies in past motion studies. Their second results suggest that first-order apparent motion inputs are processed without involving the first-order motion mechanism with respect of object tracking.

The main objective of the present study was to clarify why the upper temporal limits for object tracking by using first- and second-order motion stimuli were almost the same. To this end, we examined the temporal characteristics of object tracking by using dichoptically presented motion stimuli, as past motion studies showed that the first-order motion mechanism cannot function under dichoptic stimulation. It has been reported that, when successive frames of random-dot kinematograms are presented dichoptically, the segregation of moving areas is severely impaired (Braddick, 1974), and the apparent motions of the sinusoidal grating are difficult to detect when a stimulus is presented dichoptically (Green & Blake, 1981). Later, Georgeson and Shackleton (1989) reported that, when successive frames of missing fundamental grating patterns (square wave pattern with no fundamental frequency component) are presented dichoptically, observers perceive motion in the direction in which the physically nonexisted fundamental frequency component is shifting. These results suggest that the first-order motion mechanism is monocular and cannot detect dichoptic motion and that higher order mechanisms such as second-order motion mechanisms detect the dichoptic motion.

In the present study, we used an object tracking task similar to those used by Verstraten et al. (2000) and Kanaya and Sato (2012) with dichoptically presented motions and evaluated the upper temporal limit for object tracking. We compared our results with those obtained with ordinary binocular or monocular (nondichoptic) viewing. If similar temporal limits are obtained between nondichoptic and dichoptic stimulations, and the temporal limits are lower than those for the first-order motion detection, the results indicate that object tracking is mediated by higher order motion mechanisms without involving the first-order motion mechanism. In addition, we assume that the motion detection is a necessary component for the object tracking task used in the present study and that the upper temporal limit of motion detection determines the upper limit of object tracking. To confirm this aspect, we measured the upper temporal frequency limit for motion perception per se and compared our results with the temporal limits of the first-order motion detection and discussed motion processes involved in object tracking.

## Methods

### Participants

Five undergraduate students participated in this experiment (one female and four males, 20–22 years of age). All had normal or corrected-to-normal vision and were well trained with object tracking tasks similar to those used in the present study. The object tracking task we used is difficult for naïve participants, so we used only five really well-trained participants, who participated in numerous lengthy practice sessions. This suppressed the within-participants variation. The participants were naïve regarding the purpose of this experiment.

### Apparatus

The stimuli were generated on an Apple PowerMac G4 computer (Apple Inc., Cupertino, CA, USA) and presented on a 17-inch cathode-ray tube (CRT) monitor (FlexScan T561, EIZO NANAO CORPORATION, Ishikawa, Japan) with a resolution of 1,024 × 768 pixels and a refresh rate of 100 Hz. Participants observed stimuli through a mirror haploscope. The viewing distance (length of optical path), which was maintained using a chin rest, was 57 cm. Each pixel subtended approximately 1.8 min.

### Stimuli

The stimuli were generated using MATLAB 5.2.1 and the Psychophysics Toolbox extension, version 2.55 (Brainard, 1997; Pelli, 1997) and then presented on a CRT screen. We presented two stimulus fields subtending 11.0 deg (horizontal) × 11.0 deg (vertical) on the right and left halves of the CRT screen. The objects to be tracked (1.0 × 1.0 deg) were presented within each field. There was a black fixation dot with a diameter of 15 min at the center of each field. Two arrays containing four rectangular objects were presented sequentially to generate circular motion. All objects were placed on a circle centered at the fixation point. The distance from the fixation point to the center of each object was 3.5 deg. The second array was generated by rotating the first one by 45 deg while holding the orientation of objects upright. The two arrays were continuously alternated during each stimulus presentation, with no interstimulus interval (ISI). We fixed the ISI at zero for all viewing conditions because we did not want to have confounding between ISI and viewing condition, and it had been found that zero-ISI was one of the best conditions for the present task. The stimulus onset asynchrony of the frames varied depending on the alternation rate (temporal frequency), which was varied in five steps (from 2.78 to 5.00 Hz) for the object tracking task and in nine steps (from 1.67 to 8.33 Hz) for the motion perception task.

The object and background areas consisted of a dark/bright random-dot pattern with 50% of each type of dot. Each dot consisted of a pixel of the display, which subtended 1.8 × 1.8 min. The luminance values for dark and bright dots were 15 and 45 cd/m^2^, respectively. Therefore, the dot contrast was 0.5, and the field mean luminance was approximately 30 cd/m^2^. Furthermore, the mean luminance for luminance objects was raised relative to the background by adding a specific value (15 cd/m^2^) to the original values for both dark and bright dots. The random-dot patterns for both the object and background areas were refreshed every 20 ms.

**Figure 1.**
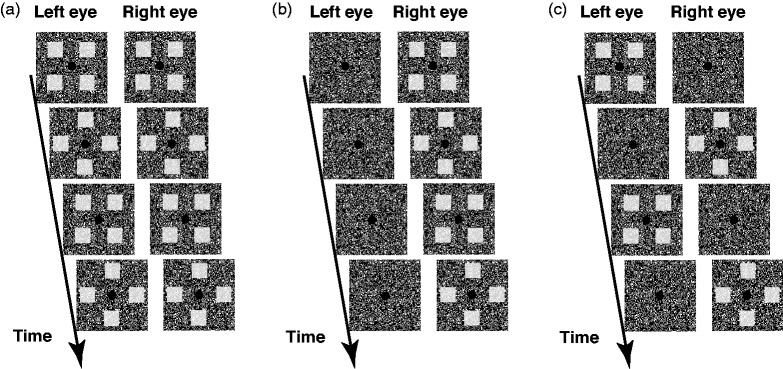
Stimulus configurations used in this experiment: (a) binocular presentation, (b) monocular presentation (presentation to the right eye), and (c) dichoptic presentation. Object and background areas consisted of random-dot patterns.

The objects were presented binocularly, monocularly, or dichoptically. Figure 1 shows the stimuli and conditions used in this experiment. In the case of binocular viewing, the same arrays of four objects were presented simultaneously to the right and left eyes (Figure 1(a)). To present the apparent motion, square- and diamond-shaped arrays were presented alternately, and the order of these (i.e., which array was presented first in a given trial) was randomized between trials. The tracking target was designated randomly from one of the objects in the first frame. In the case of monocular viewing, the arrays of four objects were presented to either the right or the left eye, and the order of the square- and diamond-shaped arrays and the target designation were randomized as for the binocular condition (Figure 1(b)). We randomized the eye to which the stimuli were presented between trials. In the case of dichoptic viewing, the two arrays of four objects were presented alternately to the right and left eyes (Figure 1(c)). The order of which eye saw the stimulus first and presentation order of the square- and diamond-shaped arrays were randomized as for the binocular condition, as well as target designation.

### Procedure

For the object tracking task, participants viewed one of the object tracking stimuli while fixating on the fixation point in a dark room. Eye movements were not monitored because tracking with voluntary eye movements in this task did not affect performance in our preliminary observations. At the start of a trial, a circular array of eight rectangles appeared, and a red rectangular marker was presented at the center of one of the eight objects to designate which object to track (target). Then, the display was switched to a motion display consisting of four rectangles with ambiguous motion. At the same time, the red marker appeared on the designated target and started to rotate in either a clockwise or a counterclockwise direction. The red marker disappeared after two circles were completed. After the marker had disappeared, participants were asked to track the target for approximately 1.8 s. Then, the alternation of the motion frames was stopped, and all eight objects were presented, with a blue rectangular marker (probe) on one of them. The participants judged whether the object on which the probe appeared was the tracked target using a two-alternative forced choice method. In half of the trials, the probe appeared at the correct position; in the other half, the probe appeared at one position before or after the correct one. Thus, the chance level of this task was 50%.

Experiments were conducted in sessions. The viewing condition (binocular, monocular, or dichoptic) was fixed within a session. In each session, the five temporal frequencies (2.78, 3.13, 3.57, 4.17, 5.00 Hz) were presented 24 times each in a random order.

For the motion perception task, we used the same stimuli as for the object tracking task and presented them for approximately 1.8 s, but no red or blue markers were presented. The participants were instructed to observe the display without tracking any particular object and to report whether they perceived rotating motion by pressing the designated key.

The experiments were conducted in sessions. The viewing condition (binocular, monocular, or dichoptic) was fixed within a session. In each session, nine temporal frequencies (1.67, 2.27, 2.78, 3.13, 3.57, 4.17, 5.00, 6.25, 8.33 Hz) were presented 24 times each in a random order.

## Results

**Figure 2.**
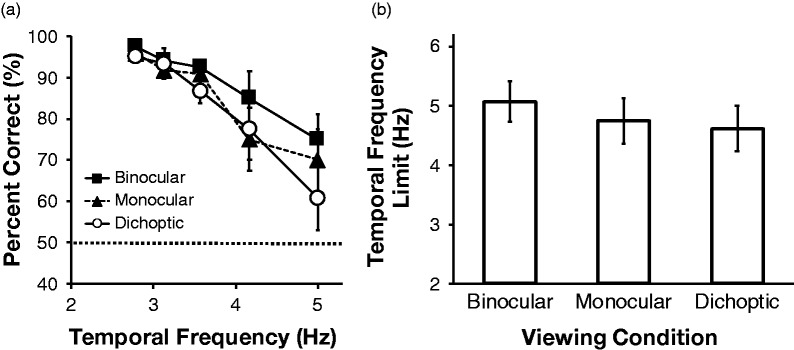
The results of the object tracking task (*N *=* *5): (a) mean percent correct scores under each viewing condition plotted as a function of temporal frequency. Error bars show ±1 *SE*. Horizontal dotted line shows the chance level (50%) and (b) mean upper temporal frequency limits of object tracking under each viewing condition. Error bars show ±1 *SE*.

Figure 2 shows the results of the object tracking task. In Figure 2(a), we plotted the mean percent correct scores from the five participants for each viewing condition as a function of temporal frequency. In general, the performance decreased as a function of the temporal frequency. We defined the limit of the upper temporal frequency of object tracking as the frequency corresponding to a 75% correct rate, which is consistent with the definition used by Kanaya and Sato (2012). We calculated the limits by fitting a logistic function to the individual tracking performance. The mean upper temporal frequency limits of the five participants under the three viewing conditions are summarized in Figure 2(b). Under all viewing conditions, the average values ranged from 4 to 5 Hz, regardless of the viewing conditions. Our one-way, repeated measures analysis of variance test indicated that the main effect of viewing type was not significant, *F*(2, 8) = 1.16, *ns*. These results suggest that the temporal characteristics of dichoptic presentations in object tracking were almost the same as those for binocular and monocular presentations.

**Figure 3.**
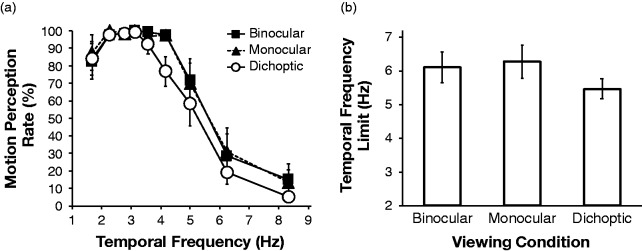
The results of the motion perception task (*N *=* *5): (a) mean motion perception rates under each viewing condition plotted as a function of temporal frequency. The error bars indicate ±1 *SE* and (b) mean upper temporal frequency limits of motion perception under each viewing condition. The error bars indicate ±1 *SE*.

We calculated the perception rate of the apparent motion for each temporal frequency and viewing condition. The results of the motion perception task are shown in Figure 3. In Figure 3(a), we plotted the mean motion perception rates of the five participants under each viewing condition as a function of temporal frequency. The perception rate was very high, up to 3 to 4 Hz. It then gradually decreased as the temporal frequency increased, reaching close to zero as the frequency increased beyond 8 Hz. Perception rates of 50% were obtained at approximately 5 to 6 Hz, regardless of the viewing conditions (Figure 3(b)). Our one-way, repeated measures analysis of variance indicated that the main effect of viewing type was not significant, *F*(2, 8) = 3.18, *ns*. These results suggest that, in the cases of both motion perception of isolated objects and object tracking, the temporal characteristics are similar under dichoptic, binocular, and monocular presentation conditions. A similar tendency with two-frame classical apparent motion was reported by Shipley, Kenney, and King (1945) for dichoptic stimulation.

## Discussion

The results of the present study can be summarized in the following two points. First, the temporal limit of object tracking for dichoptic presentation was almost equal to that for nondichoptic presentation. Second, these limits were approximately 4 to 5 Hz and were almost the same as those obtained by Kanaya and Sato (2012) for within-attribute object tracking with both first- and second-order stimuli. In addition, it was found that temporal limits of apparent motion perception (5–6 Hz) were similar to those values and again much lower than that for first-order motion detection (more than 10 Hz, e.g., Burr & Ross, 1982; Lu & Sperling, 1995b).

The assumption of this study was that first-order motion mechanism do not process dichoptic motion, but Shadlen and Carney (1986) and Carney and Shadlen (1993) proposed a quasilinear motion detector that can integrate low-level motion information from the two separate eyes. It is possible that object tracking under dichoptic presentation is mediated by this type of binocular, low-level motion detectors. However, it has been reported that these detectors have a much higher temporal frequency limit similar to that for first-order motion (10 to 60 Hz, e.g., Carney, 1997; Derrington & Cox, 1998; Hayashi, Nishida, Tolias, & Logothetis, 2007); in addition, motion stimuli these past studies used were different from our stimulus that consisted of several isolated objects. Therefore, it is hard to relate the low temporal limits of object tracking under dichoptic viewing to this type of binocular motion mechanism. Assuming that first-order motion mechanism cannot function under dichoptic stimulation, our first results imply that a motion mechanism that is different from the first-order mechanism support object tracking with dichoptic stimulation. In addition, the similarity between binocular, monocular, and dichoptic results may suggest that the motion mechanism behind these tasks is common, or at least similar.

Our second results provide some clue to identify the possible motion mechanism. The temporal limit for dichoptic object tracking obtained in this study was similar to those for within-attribute object tracking (4–5 Hz) and was much higher than those for cross-attribute object tracking (2–3 Hz) obtained in Kanaya and Sato (2012). It should be noted that the temporal limits for within-attribute object tracking were quite similar between first- and second-order stimuli. Therefore, it is likely that the motion mechanism underlying object tracking is a second-order motion mechanism that can process first-order, second-order, and dichoptic object tracking stimuli, which probably is not a complicated, higher order process that can process cross-attribute tracking stimuli.

The luminance-defined stimulus of isolated objects as used in this study has second- and higher order as well as first-order components. Therefore, both first- and second-order motion components are supposed to be extracted from the stimulus. Our results suggest that, even in the case of first-order stimuli, object tracking is performed based on only second-order components. That is, regardless of the types of motion stimuli, object tracking is mediated by a second-order motion mechanism. This hypothesis can account for the similarities among the temporal characteristics of object tracking with first-order, second-order, and dichoptically presented stimuli observed in this study.

The unresolved question is why only the second-order motion component, which leads to slower performance, is used in object tracking, even though the first-order motion component, which can be processed much faster, is available. One possibility is that, when we perceive motion of isolated object, the motion would be processed by using only the *slow* second-order motion component. Even in past motion studies, the perception of apparent motion gradually decreased as the temporal frequency increased, it was hard to perceive apparent motion beyond 5 to 7 Hz (e.g., Anstis, Giaschi, & Cogan, 1985; Kanaya & Sato, 2012; Tyler, 1973), and these temporal limits were also much lower than those of the first-order motion detection. These results are similar to our results and agree with the motion processing hypothesis about isolated objects mentioned earlier.

## Note

1 It is reported that motion detection for contrast-defined grating showed the similar temporal characteristics to that for luminance-defined grating (e.g., Lu & Sperling, 1995b). It is pointed out that these similar temporal characteristics could have been obtained because they used contrast-defined motion stimuli with static noise and the static noise affects motion detection as an artifact (Smith & Ledgeway, 1998).
